# Learning to Cooperate via an Attention-Based Communication Neural Network in Decentralized Multi-Robot Exploration †

**DOI:** 10.3390/e21030294

**Published:** 2019-03-19

**Authors:** Mingyang Geng, Kele Xu, Xing Zhou, Bo Ding, Huaimin Wang, Lei Zhang

**Affiliations:** 1 National Key Laboratory of Parallel and Distributed Processing, College of Computer, National University of Defense Technology, Changsha 410073, China; 2 National Key Laboratory of Integrated Automation of Process Industry, Northeastern University, Shenyang 110000, China

**Keywords:** multi-robot exploration, deep reinforcement learning, attention mechanism, dynamic environments

## Abstract

In a decentralized multi-robot exploration problem, the robots have to cooperate effectively to map a strange environment as soon as possible without a centralized controller. In the past few decades, a set of “human-designed” cooperation strategies have been proposed to address this problem, such as the well-known frontier-based approach. However, many real-world settings, especially the ones that are constantly changing, are too complex for humans to design efficient and decentralized strategies. This paper presents a novel approach, the Attention-based Communication neural network (CommAttn), to “learn” the cooperation strategies automatically in the decentralized multi-robot exploration problem. The communication neural network enables the robots to learn the cooperation strategies with explicit communication. Moreover, the attention mechanism we introduced additionally can precisely calculate whether the communication is necessary for each pair of agents by considering the relevance of each received message, which enables the robots to communicate only with the necessary partners. The empirical results on a simulated multi-robot disaster exploration scenario demonstrate that our proposal outperforms the traditional “human-designed” methods, as well as other competing “learning-based” methods in the exploration task.

## 1. Introduction

Exploring a strange environment as soon as possible is a classical problem in mobile robotics. It is the foundation of many real-life robotic applications such as reconnaissance, disaster rescue, and planetary exploration. In this paper, we are particularly interested in a sub-problem of robot exploration, i.e., the multi-robot exploration of a dynamically-changing environment without a centralized controller. For example, in a search-and-rescue task after an earthquake, a group of robots has to cooperate to explore the ruins and locate the survivors. Because of the instability of the building structure, the environment may be changing as time elapses, resulting in the task of each robot not being able to be statically derived in advance. As a result, the robots have to communicate with their neighbors and make decisions based both on their local views and the messages from their neighbors.

In the past few decades, the above-mentioned decentralized multi-robot exploration problem has been thoroughly studied. Various approaches have been proposed, such as the frontier-based approach and the cost-utility approach [1]. All of them are based on the cooperation strategy (i.e., the explicit communication [2] and action rules in the collective), which is “pre-designed” by humans. However, the practice has proven that many real-world settings are too complex for humans to design efficient strategies without a central node [3,4]. Besides, “pre-designed” strategies make strong assumptions about the environments and the tasks, which will limit their adaptability to dynamic environment changes and thus restrict their applications in real-world practices.

Recently, deep learning techniques have been proven to be effective solutions that target learning control policies for robotics applications [5,6,7,8]. In particular, in the multi-agent area, deep reinforcement learning has shown its great potential for multiple agents to learn the cooperation strategies alongside their policy in specific tasks, which enables the sophisticated and hard-to-design behaviors of individual agents [9]. Compared with the “pre-designed” methods, the “learning-based” approaches can automatically “learn” the complex cooperation strategies in real-world settings that are hard for humans to design in advance. Additionally, the strategies obtained by the “learning-based” approaches have shown the potential to be robust enough to handle complex and dynamic environments, and thus, will achieve better performance on the cooperation tasks than the “pre-designed” methods.

**Figure 1:**
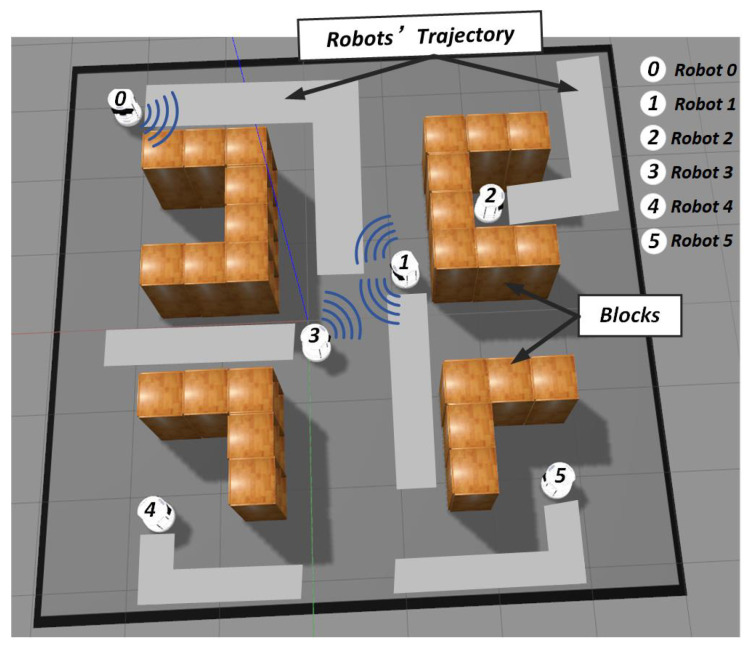
A six-robot exploration scenario; the signals stand for the correct communication method: Robot 1 should communicate with Robot 0 to avoid repeated exploration; Robot 1 should also communicate with Robot 3 to avoid target area conflict; Robot 2, Robot 4, and Robot 5 should communicate with the others less to avoid interference.

In the multi-agent area, there exist several “learning-based” methods [3,10,11,12,13,14] that model the agents’ interactions and make them learn to cooperate amongst themselves in different scenarios (i.e., cooperative navigation, cooperative push-ball, predator-prey). However, due to the special constraints of the exploration task, directly applying the existing “learning-based” methods to the exploration task is unreliable and inefficient. The reason is that, in the decentralized multi-robot exploration task, each robot should record its own historical trajectory and send the message to others in order to avoid repeated exploration. Therefore, towards a single agent, it should focus on the message from other robots without distance constraints, as long as they have explored the nearby area before. Although the existing “learning-based” methods [3,14] have made some efforts to optimize the agents’ communication process, they mainly make each agent focus more on the neighbor agents in a short distance. This inaccurate interaction modulation may make the agents neglect the message from the agents that are faraway, but which have explored the surroundings. An illustrative exploration scenario is shown in Figure 1: Robot 1 should get the trajectory message from Robot 0 to avoid repeated exploration, although they are far from each other now.

To cope with the challenges above, our work presents an Attention-based Communication neural network (CommAttn), which applies the attention mechanism to the communicating process and modulates the dynamic interactions precisely by considering the relevance between each pair of agents. Concretely, we build a Bidirectional RNN (BiRNN) [15] as the communication channel and measure each pair of agents’ communication necessity by an $O(N^{2})$ attention mechanism. Moreover, the decision-making process is completed by an RNN decoder. The inputs of the agents are fed into CommAttn by their identifier numbers, and each agent’s action is given successively in a short time interval. This will guarantee that the latter agents can acquire the previous agents’ action attempts and avoid target conflict. In order to make a quick response to the dynamic settings, we designed an exploration ratio-based training approach and gradually introduced new obstacles to the environment. This mechanism can stimulate the potential of the agents and force them to reach a specified exploration ratio with the smallest number of collisions in a limited time when faced with different kinds of dynamic environments. We demonstrate the utility of the proposed approach in a simulated multi-robot disaster exploration scenario, and the results show the superior performance of the proposed method compared with both the traditional predefined methods and the “learning-based” approach, such as CommNet (Communication neural Network) [13] and VAIN(attentional multi-agent predictive modeling) [14].

As far as we know, this is the first work that has solved the decentralized multi-robot exploration task in dynamic environments by “learning-based” approaches. The remainder of this paper is organized as follows. In Section 2, the related work is presented, with emphasis on the novelty of our work. Section 3 shows the detailed architecture of CommAttn. Section 3 and Section 4 present the details of our approach. Section 5 presents the experiments carried out on the simulation platform, as well as the analysis of the result. The discussions of our approach are given in Section 6.

## 2. Related Work

Our approach is related to two research fields, including multi-robot exploration and learning-based multi-agent cooperation. Here, we focus on the difference between existing works and our work.

### 2.1. Multi-Robot Exploration

Exploring an unknown environment by a number of autonomous robots belongs to the multi-robot exploration problem, whose the goal is to explore a strange environment as soon as possible by the cooperation among multiple robots. Plenty of efforts have been made on the multi-robot exploration problem. The exploration problem can be divided into two kinds: exploring a static environment and a dynamic environment.

For static environments, early works often solved the problem by geometric methods, such as frontier-based methods [16] and market-based methods [17]. The coordinated frontier-based approach uses a simple agent-frontier assignment algorithm detailed in [18]; in short, every robot determines frontier utilities for itself and its nearby teammates and iteratively calculates a robot to the frontier assignment that maximizes the joint utility. The nearest frontier approach is based on Yamauchi’s technique [16], and it consists of selecting the shortest path to the nearest frontier. As can be noticed, this method does not consider the utility of the frontiers and the coordination mechanism. Therefore, this method can save time on frequently re-planning the target area. The cost-utility approach [19] introduces a method of information gain that measures the utility of reaching a given cell. The frontier cells are designated as candidate destinations, and the potential selection is based on both the travel cost and the utility. For market-based methods, the robots place bids on sub-tasks of the exploration effort. These bids are typically based on values such as expected information gain and travel cost to a particular location in the environment. Recent approaches focused more on mutual information for ranging sensors [20,21], and they attempted to maximize mutual information [21,22,23,24] directly. None of the approaches above take the dynamic change of the environment into account, which is different from our goal, i.e., to cope with a dynamically-changing environment.

For dynamic environments, a Real-time Auction-based Dynamic Allocation Scheme (RTABA) [25] was proposed to explore a set of targets with minimization of the total cost objective. They dealt with the dynamic and uncertain environments by means of incremental assignments based on up-to-date situations of the environment. This method can eliminate the redundant calculations and save the time for frequent target re-allocations. Another work solved the cooperation by focusing on rules for determining bids affecting the performance of the auction mechanism, establishing bounds on the performance of different rules [26,27]. Later work considered the sequential single-item auction [28,29] as an alternative to auctions for multi-robot routing. A method [30] based on different evaluation functions was proposed to develop successful control policies for dynamic and stochastic multi-robot exploration missions. The methods above are all based on the “pre-designed” cooperation strategy, and they should take all the uncertainties into account to cope with the dynamic environments. Additionally, the “pre-designed” strategies make strong assumptions on the environment and the task features, which will limit their applications in real-world practice because of the low adaptability to different task environment features. In many real-world settings, with multiple agents with partial observation ability, it is extremely hard for humans to design effective strategies due to the local view points of agents [31]. In our method, we bypass the complex cooperation strategies’ design process and make the agents learn to cooperate amongst themselves.

### 2.2. Learning to Cooperate Based on Explicit Communication

Learning cooperation strategies in multi-agent systems by communication methods could be realized by implicit and explicit communication. Here, we focus on explicit communication, which is a specific act designed solely to convey information to other robots on the team. The collaboration of multiple agents by explicit communication is usually realized by two kinds of methods: “pre-designed” (manually specified) and “learning-based” methods. Typically, in the “pre-designed” methods, the specification and format of the communication are pre-determined, i.e., in robot soccer, the agents are designed to communicate their position and proximity to the ball at each time-step [13]. However, in the “learned” methods, what each agent transmits is not specified prior, being learned instead. In addition, the interpretation of the received message is also learned by the agents to get a more robust communication protocol, which will be hard for humans to design. In other words, the “learning-based” methods can make agents learn a communication that aids the performance alongside their policy. There are mainly six end-to-end trainable models that have been proven effective to learn to cooperate: Communication Neural Network (CommNet) [13], Differentiable Inter-Agent Learning (DIAL) [10], Bidirectionally-coordinated nets (BicNet) [11], Multi-agent Deep Deterministic Policy Gradient (MADDPG) [12], Vertex Attention Interaction Network (VAIN) [14], and Supervised Attention-based Message Processing (SAMP) [3]. The first four approaches were proposed without applying the attention mechanism to the communicating process. The last two approaches use different attention mechanisms to optimize the communication process in different scenarios.

CommNet [13] uses a single network in the multi-agent setting by passing the averaged message over the agent modules between layers. CommNet tries to obtain an integrated communication vector for each agent by averagely pooling over all messages broadcast from the agents. However, a significant drawback is not explicitly modeling the interactions and putting the whole communication burden on the message extractor. DIAL [10] was introduced to solve simple communication tasks, but it could not solve the problem in non-stationary environments. BicNet [11] was proposed to handle real-time strategy games such as StarCraft. However, it assumes that the agents are fully observable for the environment, which is not realistic in practice. MADDPG [12] extends the traditional actor-critic methods to the multi-agent coordination area. However, it solves coordination by directly introducing other agents’ observations and actions. This may lead to the problem of excessive state space and could not be applied to large-scale multi-agent environments.

VAIN [14] uses the attention mechanism to improve communication efficiency. In order to improve the average pooling mechanism of CommNet, VAIN introduces attention vectors to model the distinct interactions between agents with a time complexity of O(N). Although the interactions are able to be modulated, VAIN can hardly update the dynamic interactions for the latter agents based on the previous agents’ output states. In addition, in most cases, VAIN just focuses on the agents in a close range, which would not be suitable for the exploration task. The reason is that an agent’s action in the exploration problem should be influenced by whether the area around its current location has been explored. However, the historical trajectories are only recorded by the agents themselves, and their positions vary over time.

SAMP [3] uses supervised signals to optimize the attentional weights with a target auxiliary interaction matrix from the environment. The results outperform other competing multi-agent methods in the “predator-prey-toxin” methods domain. The idea behind the supervised signal is straightforward, that they want to strengthen the interaction $a_{ij}$ when agent *i* and agent *j* get rewards. However, this auxiliary information can be thought of as an intrinsic reward [32], which is task specific and needs appropriate construction according to the task specification. Essentially, for the exploration task, the situation that both agent *i* and agent *j* get positive rewards does not specifically mean they should focus more on each other. The reason is that the individually-corrected decision makings of two agents at a far distance from one another will also lead to two positive rewards. Therefore, it is hard to design such a supervised signal in the exploration task. In this paper, we focus on a precisely-weighted mean of the agents’ internal states and propose CommAttn by applying the attention mechanism with time complexity $O(N^{2})$ to model agents’ interactions by considering the relevance of each received message. For each agent’s output state, we precisely measure its correlation with every agent’s input state. Thus, we can integrate more comprehensive information for each agent’s decision-making process.

## 3. Methodology

**Figure 2:**
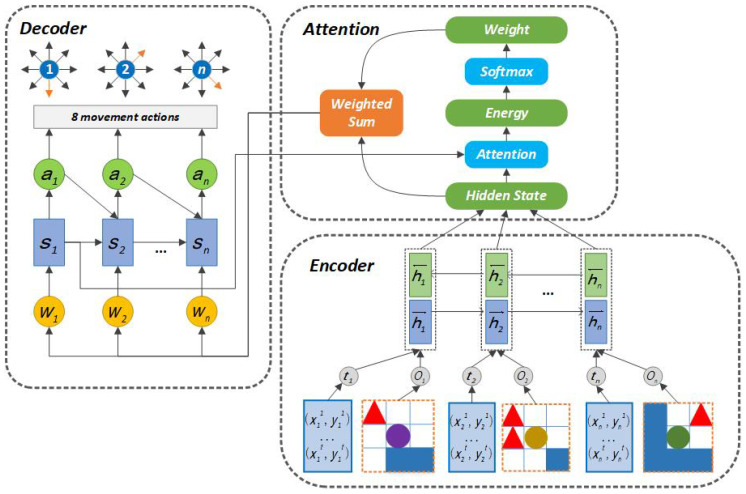
The architecture of the Attention-based Communication neural network (CommAttn): each agent’s input is composed of two parts: the local observation (the environment knowledge within the agent’s vision range) and the trajectory (a set of the agent’s history positions).

In this section, we will introduce our designed communication model CommAttn, which can selectively retrieve the most valuable information for each agent to make decisions in the exploration task. We first explain why CommAttn can be applied to the exploration task from theory. We aim to clarify two questions: how to maintain each agent’s historical trajectory and how to choose the most valuable messages for each agent. In order to make the exploration task easy, we abstract the robots as agents and assume that the locating problem has already been solved. Therefore, each agent can record its trajectory by storing the history positions. Every time it moves, it will add its current location to the end of the list. Then, the trajectory list and the local observation will be sent to the communication channel. For the second question, we choose a Bidirectional RNN (BiRNN) [15] as the communication channel, from which each agent can get the other agents’ messages. Each agent can selectively retrieve the most valuable messages from the channel based on a weight calculation layer (the energy part in Figure 2. Each time CommAttn generates an agent’s action, it searches for a set of agents where the most relevant information is concentrated. Then, CommAttn predicts the next agent’s action based on the integrated vectors from the communication channel associated with the selected agents and all the previous agents’ actions. Based on the information above, each agent can learn how to express its state, selectively understand the broadcast information from other agents, and finally learn to cooperate.

We then describe the architecture of CommAttn. As shown in Figure 2, CommAttn is composed of three parts: encoder, attention, and decoder. The encoder part uses a BiRNN as a communication channel to maintain the agents’ initial internal states and outputs an integrated vector. The attention mechanism maintains the ability to model agents’ interactions by taking the relevance of each pair of agents into account. Towards each agent, the attention mechanism can calculate how much other agents’ inputs will affect its decision-making process. The decoder function can analyze the weighted information (the dot product of attention weights and each agent’s hidden state) and output the actions for each agent.

A BiRNN is composed of forward and backward RNNs. The forward RNN $\vec{f}$ and backward RNN $\overset{\leftarrow }{f}$ read the inputs (each agent’s local observation together with the trajectory) in forward and reverse orders respectively in order to get the corresponding hidden states ($\vec{h_{1}},\cdots ,\vec{h_{n}}$) and ($\overset{\leftarrow }{h_{1}},\cdots ,\overset{\leftarrow }{h_{n}}$). Here, we denote *n* as the number of agents. Then, we obtain the integrated hidden state $h_{i}$ for each agent *i* by concatenating the forward hidden state $\vec{h_{i}}$ and the backward one $\overset{\leftarrow }{h_{i}}$.

The model used in the decoder process is an RNN layer. Each agent’s action is given by:

(1) $$\begin{array}{c} p\left(a_{i}|a_{1},\cdots ,a_{i-1}\right)=g\left(a_{i-1},s_{i},c_{i}\right), \end{array}$$

where $s_{i}$ is an RNN hidden state for time *i*, computed by:

(2) $$\begin{array}{c} s_{i}=f\left(s_{i-1},a_{i-1},c_{i}\right), \end{array}$$

and the corresponding input $c_{i}$ is a weighted sum of each agent’s integrated hidden state in the encoder process:

(3) $$\begin{array}{c} c_{i}=\sum_{j=1}^{n}\frac{exp(e_{ij})}{\sum_{k=1}^{n}exp(e_{ik})}h_{j}, \end{array}$$

where:

(4) $$\begin{array}{c} e_{ij}=m\left(s_{i-1},h_{j}\right), \end{array}$$

*m* is a matching model that scores how well the agent’ observation around position *j* and the action of agent *i* match ($e_{ij}$ of the energy function in Figure 2). The matching model is a feed-forward neural network that can be jointly trained with the other parts. Due to the matching of each pair of agents, the complexity of CommAttn is $O(N^{2})$. Although this complexity is larger than the one of VAIN O(N), CommAttn can reduce the consumed time by explicitly modeling the interactions and is suitable enough for the exploration task.

The method of taking a weighted sum of all the agents’ integrated hidden states can be regarded as computing an expected hidden state. The purpose is to average only information from relevant agents (e.g., particularly influential agents). The weights measure the importance of interacting among agents. In other words, the decoder decides which parts of the source observations should be focused on and relieves the encoder from the burden of compressing all the useful information.

The model is trained by the policy gradient [33] algorithm whose gradient comes from a state-specific baseline b(s,θ). In an episode of length *T*, towards a single agent, the states are represented as s(1),⋯,s(T), and the actions are represented as a(1),⋯,a(T). Then, for each agent *j*, the loss function can be calculated by:

(5) $$\begin{array}{c} loss_{j}=\sum_{t=1}^{T}\left[\log p\left(a\left(t\right)|s\left(t\right),\theta \right)\left(\sum_{i=t}^{T}r\left(i\right)-b\left(s\left(t\right),\theta \right)\right)-\alpha {\left(\sum_{i=t}^{T}r\left(i\right)-b\left(s\left(t\right),\theta \right)\right)}^{2}\right]. \end{array}$$

The loss of the decoder part is the sum of all the agents’ loss functions. The optimizing purpose aims not only to maximize the expected reward, but also to minimize the distance between the baseline and the actual reward. Here, r(t) is the reward given at time *t* and α is for balancing the baseline objectives and the reward.

## 4. Implementation Details

In this section, we will describe the details of our approach in applying CommAttn to the exploration task. Our approach is composed of three aspects. Firstly, we constructed the modeling of dynamic environments on the basis of the occupancy grid [34]. Secondly, we devised appropriate reward functions with the aim of encouraging the robots to learn the ability to cooperate. Finally, we designed the exploration ratio-based training technology for the exploration task.

### 4.1. Modeling of the Exploration Environment

The exploration environment was represented by occupancy grids. The basic idea behind it is to represent the map as a two-dimensional grid of binary random variables, which stand for whether the locations are occupied.

We give some definitions first: $z_{t}$ stands for the observation of the robot; $c_{t}$ stands for the communication message from other robots within the certain range; $a_{t}$ stands for the output actions given the corresponding inputs; π($a_{t}\left|z_{1:t},c_{1:t}\right)$ stands for the policy for choosing controls based on the past observations and communication message.

For a better explanation, we assume that there is an underlying grid map $m\in M={\left\{0,1\right\}}^{N\times N}$ primarily unknown to the robots. Each robot wishes to calculate its belief over maps *M* at time *t* given all its previous observations and other robots’ communication message leading up to that time-step $b_{t}\left(m\right)=p\left(m|z_{1:t},c_{1:t}\right)$. To simplify the problem, we assume the individual map random variables, indexed as $m_{i}$, are independent:

(6) $$b_{t}\left(m\right)=\prod_{i}p\left(m|z_{1:t},c_{1:t}\right)=\prod_{i}b_{t}\left(m_{i}\right).$$

The robots learn to update the posteriors themselves. The information-theoretic [34] entropy of the belief state is used to quantify the uncertainty, which factorizes over the individual map random variables because they are assumed to be independent:

(7) $$\begin{array}{cc} H(b_{t}\left(m\right)) & =\sum_{i}H\left(b_{t}\left(m_{i}\right)\right) \end{array}$$

(8) $$\begin{array}{c} =\sum_{i}b_{t}\left(m_{i}=1\right)\log b_{t}\left(m_{i}=1\right)+b_{t}\left(m_{i}=0\right)\log b_{t}\left(m_{i}=0\right) \end{array}$$

### 4.2. Entropy-Oriented Reward Function

Now, we describe the approach to devising the reward functions. At each time-step *t*, the agent receives an observation $o_{t}$, which is composed of the agent’s local view and its trajectory. At this point, the agent is faced with the decision of what action to select given the observation. In our RL (Reinforcement Learning) formulation, each agent seeks a policy that reduces uncertainty for the whole map as quickly as possible. The reward function of each agent can be divided into two kinds: the individual reward and the mean global reward. Therefore, at each step *t*, an agent gets the following reward:

(9) $$R_{t}=B^{back}r_{back}+C^{t}r_{coll}+B^{final}is_{succeed}r_{succ}+B^{final}\left(1-is_{succeed}\right)r_{fail},$$

where $R_{t}$ is chosen to force the agent to explore a new area and avoid collisions with other agents at time-step *t*. Here, $B^{back}$ is a Boolean flag that judges if the agent reaches an area that has already been explored by the team. $r_{back}$ is fixed to −10 in order to punish the useless exploration. $C^{t}$ refers to the number of collisions (blocks or other agents) with the current agent. Two agents collide if their locations overlap. A collision with other agents or the blocks incurs a reward $r_{coll}=-10$, but does not affect the simulation in any other way. The above part stands for the individual reward, which is designed to force the robots to explore a new area and avoid collisions with blocks and other robots. The mean global reward is applied only on the last time-step with the aim of guiding the team to learn to cooperate. The simulation is terminated after the specified steps, and the standard of success is specified according to the difficulty of the task.

Here, $B^{final}$ judges whether this is the last time-step. $is_{succeed}$ stands for whether this episode is judged as a success. Concretely, at the last time-step, each robot will receive a mean global reward, which stands for the cooperative ability of the teams. Concretely, if the exploration ratio of the team achieves a certain standard, then each agent will receive a reward $r_{succ}$ of 40. Otherwise, each agent will get punished by a reward $r_{fail}$ of −5. This will further stimulate the potential of the whole team to improve their cooperative ability. We can then train a policy to maximize the expected reward in simulation and improve the whole system’s cooperative ability.

### 4.3. Exploration Ratio-Based Training Approach

The task was trained for 50 epochs, each epoch being 100 weight updates on a mini-batch of 288 game episodes (distributed over multiple CPU cores). The training time took about five days on our 24-GB-memory server. To make the learned strategy robust to dynamic settings, we gradually added new blocks to the original environment (*n* blocks every *m* time-steps). Each simulation was terminated after a specified number of time-steps and classified as a failure if collisions with blocks had occurred or the exploration ratio $el_{ratio}$ was less than 90%. $el_{ratio}$ here is calculated as follows:

(10) $$el_{ratio}=\frac{count(S_{explored}⋃S_{finalblock})}{A},$$

$S_{explored}$ being the subset of explored cells in the map, $S_{finalblock}$ the subset of final blocks, *A* the number of cells in the map, and count(X) a function that counts the number of elements in the set *X*. Here, we take the operation ⋃ because the positions of the newly-generated blocks may overlap with the area that has been explored by the multi-robot system. Then, the network can optimize the weights to increase the success rate of the whole system.

## 5. Experimental Results

**Figure 3:**
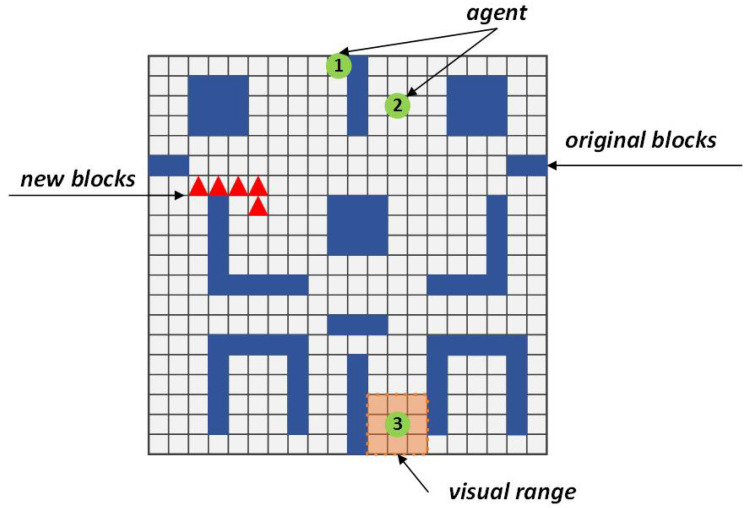
The experimental environment, which is dynamic in the number of blocks.

In this section, we implement a series of experiments to validate the effectiveness of CommAttn and demonstrate the advantages over “pre-designed” methods and other competing “learning-based” approaches. We used MazeBase [35] to set up an environment to test our approach. As shown in Figure 3, the map represents an artificial environment with many bifurcations and loops on a 20×20 grid, in which new blocks can be introduced randomly at any time. We discretize the locations of the agents so that each of the agents is in a single position in the grid. The agents can sense the information in the adjacent positions within their vision range (a surrounding v×v neighborhood). The agents can move to eight adjacent positions that do not contain blocks. Each agent can explore one cell in one step. The goal of the multi-agent system was to reach the specified exploration ratio with the smallest number of collisions in a given time-step.

We will introduce our experiments from the following three aspects: the results compared with the “pre-designed” strategies, the results compared with the “learning-based” methods, and the corresponding analysis. The experiments compared with the “pre-designed” strategies aimed to highlight the short response time and the robustness of the “learned” strategies to cope with the dynamic environments. Similarly, the experiments compared with the “learning-based” methods were intended to show the suitability of our attention mechanism for the decentralized multi-robot exploration task. Furthermore, to understand the nature of interactions between the agents to cope with different scenarios, we give the visualization of the communication process.

### 5.1. Results Compared with the “Pre-Designed” Strategies

In order to demonstrate the superiority of CommAttn in dealing with the dynamic environments compared with the “pre-designed” strategies, we evaluated three competing traditional methods as baselines. The three “pre-designed”methods were the coordinated frontier-based approach [18], the nearest frontier approach [16], and the cost-utility approach [36].

**Figure 4:**
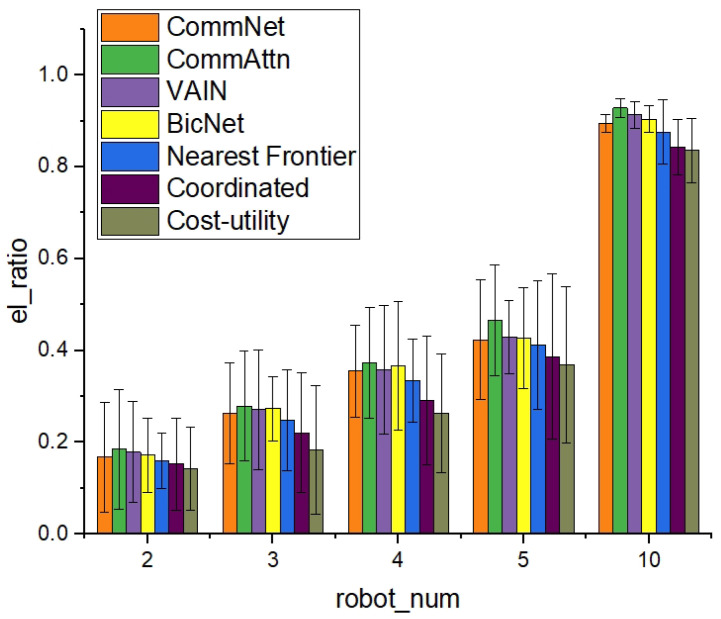
The exploration rate in 30 s of a different number of robots.

We conducted the experiments in static and dynamic environments, respectively. In the static environment, we compared the “learned” methods (CommNet [13], VAIN [14], and BicNet [15]) with the three “pre-designed” methods and focused on the index of the number of covered cells in the given time. As shown in Figure 4, the “learned” strategies obtained better performance on the exploration speed. The efficiency of the exploration speed can be attributed to the quick response of the neural network and the high quality of decision making. As the number of mobile agents increases, the exploration time will decrease due to the abundant resources and task allocation. The goal of CommAttn was to minimize the repeated exploration by the learned cooperation strategies and make each agent explore new cells in each decision-making process. From the experimental results, the number of robots did not affect the performance difference too much in each of the different methods. For the traditional “pre-designed” methods, our results were consistent with the results reported in [1]. Concretely, the performance of the “nearest frontier” approach was always better than that of the “coordinated” approach. The performance of the “coordinated” approach was always better that of the “cost-utility” approach. For the learning-based methods, the learned strategies stand for the coordination ability of the agents. Therefore, the more robust the strategies are, the more efficient performance the robots will perform regardless of the number of robots.

In order to provide a demonstration, we compared the planning time in the decision-making process of the four approaches. As shown in Table 1, the response time of the CommAttn is much less than those of the “pre-designed” strategies. The planning time of CommAttn is one-eighth of that in the cost-utility approach and nearly the same as that of the nearest frontier approach. However, the high-quality decision making was much more optimal than the nearest frontier approach. In particular, in the extremely complex environments, i.e., plenty of loops and circuits, CommAttn’s performance was superior by avoiding frequently getting into the local dilemma.

As for the dynamic environment, we measured the relationship between the exploration ratio $el_{ratio}$ and the adding frequency of blocks in the given time-steps. The survey region contained 70 newly-introduced blocks, which will be added according to a uniform random distribution across the search space as the exploration task progresses. Concretely, only 81 static blocks are active at the beginning; as the mission progresses, more blocks become active at a rate of *n* blocks per *m* time-steps, such that all blocks are active after 90% of the mission time. In each test, the approaches were faced with the same obstacle generating strategy. Although the adding frequency of blocks was changing over time, the total number of newly-introduced blocks never changed, with the aim of a fair comparison.

**Figure 5:**
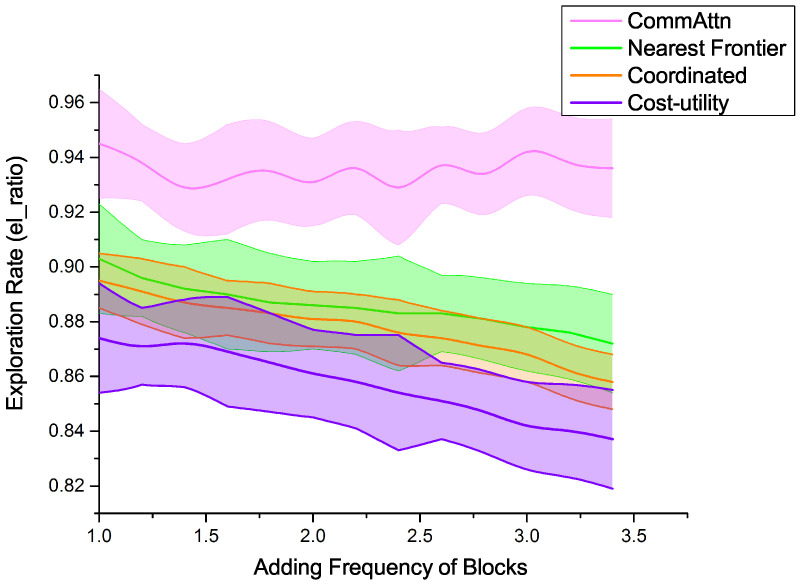
As the adding frequency of the blocks increases, CommAttn shows a more stable exploration efficiency than the baseline “pre-designed” methods.

From Figure 5, we can see that, as the adding frequency of blocks increased, CommAttn performed largely better and more stably than the baseline methods, and this suggests that CommAttn is robust enough to be adapted to more complicated environments. Compared with the other three “pre-designed” strategies, CommAttn performed better on the exploration efficiency and the ability to adapt to changing environments. This suggests that CommAttn could obtain more robust strategies than the “pre-designed” methods.

We now explain why CommAttn shows more stable performances in the dynamic environments compared with the “pre-designed” approaches. The “pre-designed” strategies are so complicated that this increased the planning time of the whole system. Concretely, the agents needed to complete several complicated processes: target selection, auction negotiation process, task exchange mechanism, and the precautions, which can all be substituted with a robust strategy learned by CommAttn and save the corresponding time. CommAttn maintains the ability to make a quick response to the dynamic environments online based on current knowledge. In other words, the targets are dynamically assigned to the agents in each step. This is achieved by forcing the team to achieve a specified exploration rate at a fixed time. After plenty of training epochs specified by a random distribution of the dynamic blocks, the agents can master the skills that can make them suitable for different kinds of uncertainties. The experimental results also demonstrated that this training method can stimulate the potential of the whole team. Therefore, although the environment is dynamic, the agents can learn more robust strategies and are able to deal with the sudden changes by taking the most valuable reactive actions. In addition, the actions produced by the “pre-designed” methods were sub-optimal compared with CommAttn. In CommAttn, the agents can make a quick response to the dynamic environments and adjust to the best target area from the current state.

**Figure 6:**
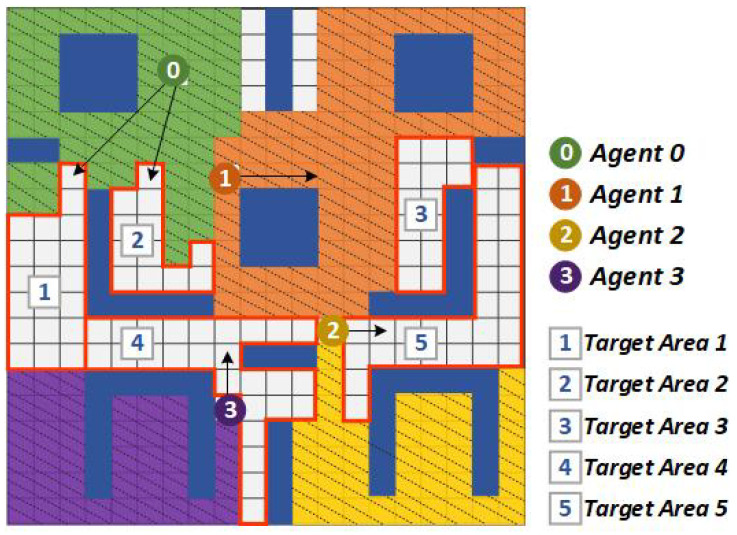
An illustrative scenario (the initial environment and the corresponding actions for all agents) to show how CommAttn successfully deals with dynamic environments (newly-introduced blocks).

**Figure 7:**
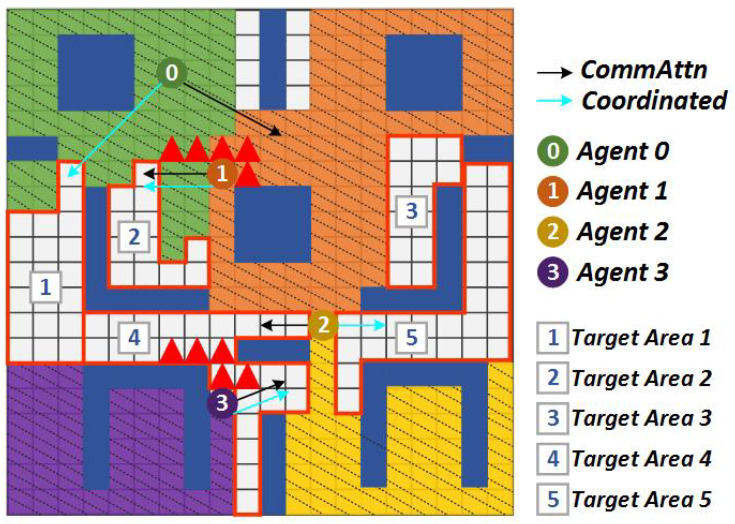
The optimal actions of CommAttn and the sub-optimal actions of the coordinated frontier-based approach after some unexpected blocks introduced to the environment.

An illustrative example of the comparison between CommAttn and the coordinated frontier-based approach is shown in Figure 6 and Figure 7. In this scenario, there were five remaining target areas that had not been explored and four robots in the environment. Here, we denote $r_{a}:t_{b}$ as the *a*th robot allocated with the *b*th target area. From the estimated distances among targets and agents, $r_{0}:t_{1},t_{2}$, $r_{1}:t_{3}$, $r_{2}:t_{4}$, and $r_{3}:t_{5}$ assignment gave the lowest total cost of 13. However, when the obstacle was suddenly detected (Figure 7), the primary routes of $r_{1}$ and $r_{2}$ were blocked. As a result, CommAttn changed the allocations to $r_{0}:t_{3}$, $r_{1}:t_{1},t_{2}$, $r_{2}:t_{5}$, and $r_{3}:t_{4}$, which was the best solution confronted with the current state. In contrast, the coordinated frontier-based approach changed the allocation to a sub-optimal one. Therefore, CommAttn can be well suited to dynamic environments by making the best-coordinated solution online.

### 5.2. Results Compared with the Existing “Learning-Based” Methods

**Figure 8:**
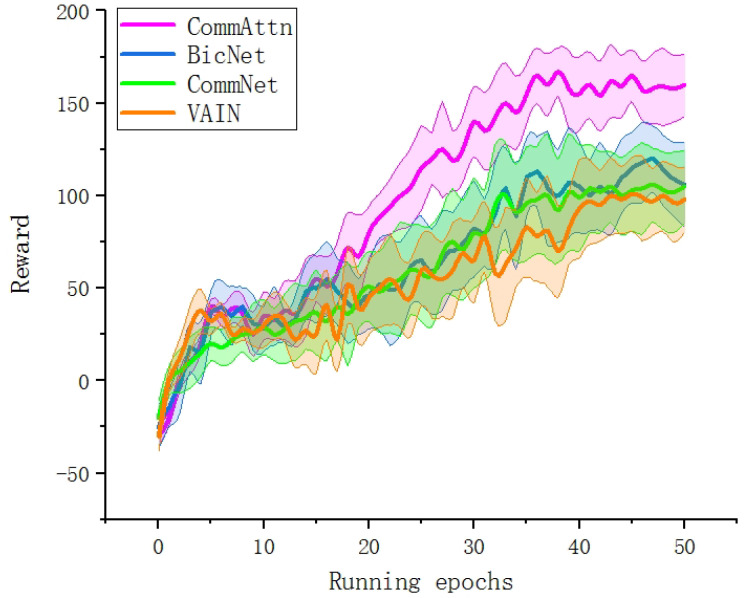
The variation of the agents’ summed scores in the training process between CommAttn and the baseline “learning” methods.

To examine the effectiveness, we first focused on the comparison between CommAttn and the “learning-based” methods (BicNet, CommNet, and VAIN). We did not take MADDPG into account because MADDPG directly uses the states and actions of all other agents for cooperation instead of utilizing communication. Therefore, MADDPG can hardly adapt to the decentralized multi-robot exploration task. The performance of each approach was assessed by the sum of the rewards of all the agents (also called the score) in each episode. Ideally, each agent predicts the actions of other agents based on its local observation and the received information from others and completes its own decision-making process. As shown in Figure 8, CommAttn outperformed all the baselines in the score and reached roughly 150% of the score acquired by the other three models. This indicates that CommAttn has a better learning ability in the exploration task, which could not be mastered by the three baseline methods.

Additionally, we conducted an analysis of the cooperation strategies that CommAttn has learned. As the training process goes on, CommAttn would be able to discover effective collaboration strategies. In the initial stages of learning, the agents could not obtain the ability to avoid colliding with each other or blocks. They are more likely to target their own nearest area, and this may cause collisions. With the increasing rounds of training, the agents could master the skills to focus on different parts of the map. When they have the potential to reach the same target area, they will communicate effectively and then disperse to different areas. Finally, when the training becomes stable, a reasonable allocation of target areas could be realized. Concretely, the agents can disperse in their target areas before they gather into crossings and reach an agreement beforehand.

**Figure 9:**
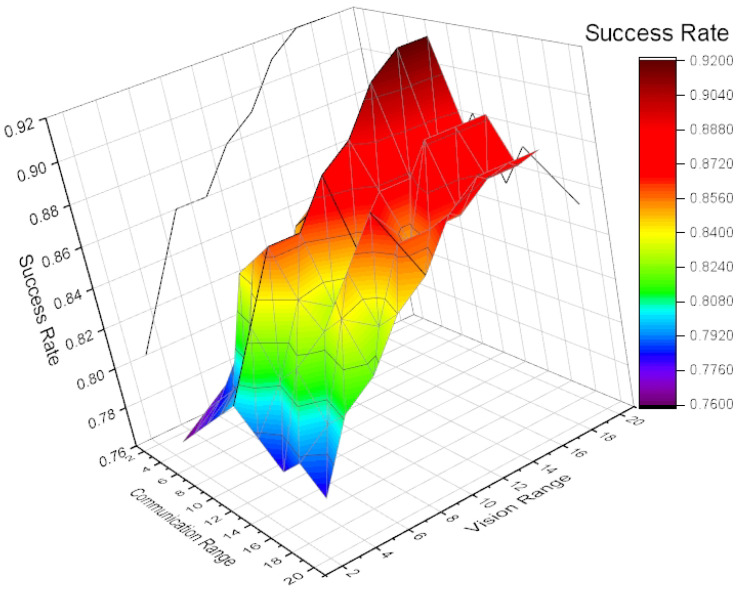
The relationship among the success rate, the vision range, and the communication range of CommNet.

In order to demonstrate the effectiveness of the $O(N^{2})$ attention mechanism and validate the supposition that massive information will mislead the agents’ decision-making process, we explored how the vision range and communication range affected the exploration efficiency. We specified the two variables and implemented the exploration task 1000 times. As shown in Figure 9, the average reward of all the agents was proportional to the vision range. This suggests that the agents can successfully deal with the observation acquired by themselves. The observation just contains the relative locations of the other agents and blocks excluding the message from others. However, the success rate fluctuated when the communication range was larger than 11. This validates our supposition that when the communication group is large enough, the agents will suffer from being overwhelmed by massive received messages. This can make the agents have difficulties in acquiring valuable information. Therefore, an accurate attention mechanism is necessary for the agents to select the most meaningful information that will help them make decisions.

We evaluated CommAttn and the baselines by running 1000 episodes and compared the average mean reward, the number of collisions, and the exploration rate $el_{ratio}$ at the end of each episode. Each episode lasted for 30 s. As shown in Table 2, CommAttn significantly outperformed the baselines. The major reason is that CommAttn acquired the ability to select the most valuable message and drop the useless information, which may interfere with the agents’ decision-making process. The main strategy of CommAttn is that an agent is first attempting to reach the nearest unexplored cells. The way to determine whether the target area has been explored by other agents is realized more accurately by getting a precisely-weighted vector from all the agents. Similarly, if the target area is more likely to be occupied by the other agents, the agent will turn to another target cell instead of continuing to probe and wasting time.

In contrast, the strategy of CommNet is more conservative. For example, it prefers to avoid collisions with other agents and neglects whether the target cell has been explored by other agents. This eventually leads to a low exploration rate $el_{ratio}$ in the fixed time. Moreover, CommNet agents are more willing to gather together and focus on other agents’ assumptions. VAIN performed slightly better than CommNet. An agent in VAIN usually focuses more on the message from nearby agents. However, if a cell near the current agent has been explored by an agent who is now far away, the agent could not capture this valuable information in a timely manner. This is mainly caused by the O(N) complexity of the attention mechanism, which is much more simple and cannot clearly reveal the interactions between each pair of agents. The strategy of BicNet is more aggressive, i.e., the agents usually approach their nearest target area. Therefore, when multiple agents choose the same target cell, then they are prone to collisions.

The baseline methods also have communication. Now, we demonstrate the reasons why their performances were much worse than CommAttn. CommNet simply averages the information of all the agents’ hidden layers. This operation just applies equal focus on all the other agents. However, the information from other agents should have different priorities towards a single agent’s decision-making process. For example, the information from the agent who attempts to reach the same target area is much more valuable than the agents who will not collide with the current agent. At the initial exploration stage, when an agent is exploring an area far from all the other agents, there will be much meaningless information, which can be regarded as noises that will affect the agents’ decision making. VAIN introduces an attentional vector to measure the communication strength of an agent and then weights the interaction between each pair of agents by a kernel function. The complexity of this attention mechanism is O(N). Therefore, it could not precisely reveal the relationship between each pair of agents. This will cause the problem that an agent can not pay enough attention to the agents that are far away. Therefore, if the agents at a long distance have explored the surrounding area of the current agent, the useful information could not be mastered in a timely manner. Regarding common issues related to each of the methods, the traditional “pre-designed” methods heavily relied on the human-designed cooperation strategies, which will restrict the potential for the whole group to learn more robust strategies to deal with the uncertainties in a dynamic environment. The existing learning-based methods do not design an approximate communication architecture to solve the multi-robot exploration problem, which needs precise employment of the trajectory information from each robot in the communication process. In CommAttn, inspired by the success of the attention mechanism in seq2seq [37], we selectively attended to specific information from other agents according to the decoder state. Moreover, the latter agents’ actions can be updated in a timely manner by the previous agents’ output states. Concretely, from a macro perspective, the agents make decisions simultaneously. However, from a microcosmic perspective, the agents make decisions in succession; this will guarantee that the latter agents can get the previous agents’ actions and avoid target conflicts.

### 5.3. Analysis of Communication

**Figure 10:**
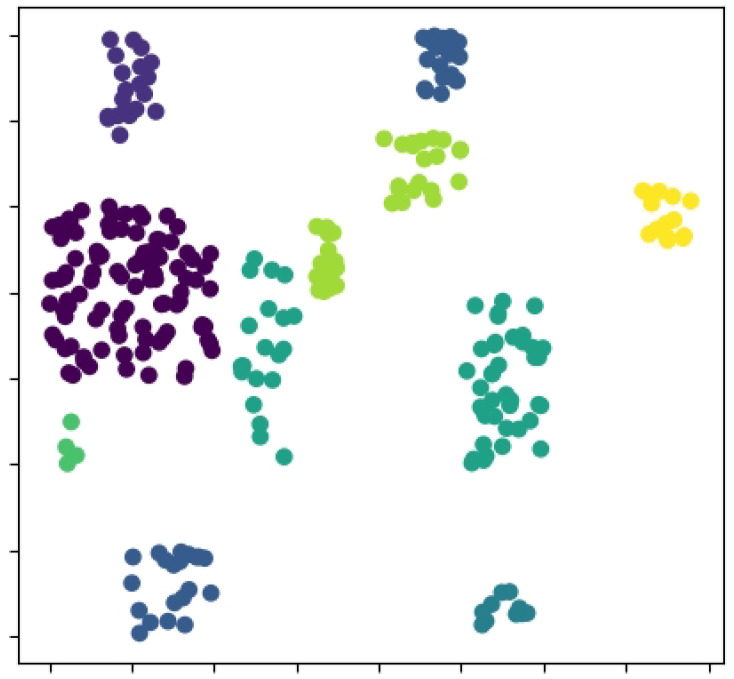
The values of the hidden state $s_{j}$ of each agent from the decoder part in the static exploration environment.

**Figure 11:**
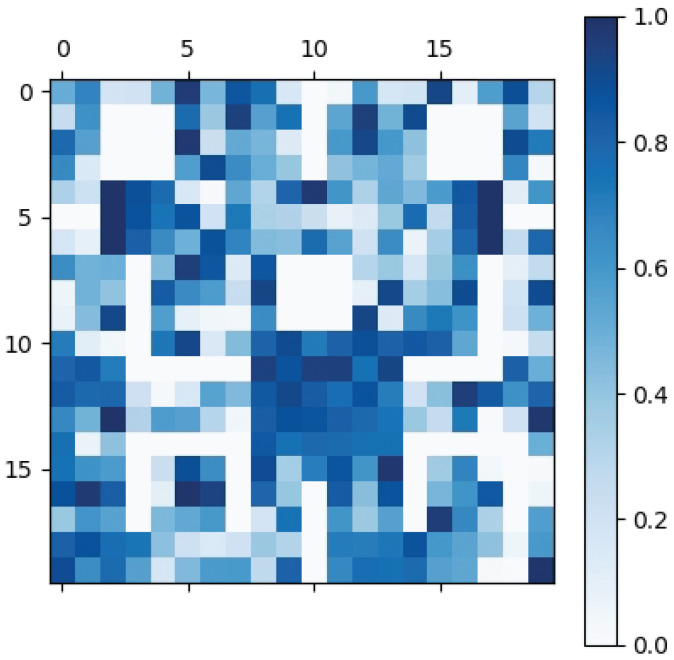
The average norm of communication vectors in the static environment.

As when and what to communicate in CommAttn are spontaneously generated by the agents themselves, we now attempt to understand what the agents communicate themselves while performing the exploration task. We start by recording the values of the hidden state $s_{j}$ of each agent from the decoder part. Figure 10 shows the t-SNE (t-distributed Stochastic Neighbor Embedding) [38] of the hidden state. Distinct clusters are clearly present, which indicates that the agents can coordinate themselves when necessary (the target area is in conflict). We also visualize the average norm of the communication vectors in Figure 11 over the 20×20 grid without newly-introduced blocks. We can see that the agents have a stronger communication intention on the important positions such as the connection area between blocks, which indicates the effectiveness of CommAttn.

## 6. Conclusions

In this work, we introduced CommAttn to train a decentralized multi-robot system to learn cooperation strategy in the exploration of dynamic environments via an $O(N^{2})$ attention mechanism. By using the multi-agent reinforcement learning technique and deliberately-designed reward functions, the robots can learn to communicate with each other, making decisions based on the communication messages and their states, and thus seamlessly cooperate in the multi-robot exploration process. The communication model learned by the multi-robot system is robust enough to adapt to the dynamic changing of environments. Experimental results show that the cooperation strategy gained by CommAttn achieved a performance better than the traditional “pre-designed” strategies and the competing multi-agent communication models.

This work is preliminary in that it only evaluated a simulated scenario. In future work, we hope to apply this approach to problem setups with limited communication bandwidth and noises. Concretely, we will try to solve the problems (i.e., localization, noisy observation problems) by exploiting the hierarchical arrangement of communication policies and local agent policies. We believe that our framework will effectively be applied to real-robot systems by designing the approximate mechanism to deal with the noise problem in realistic settings and lead to a fully-trainable exploration system.

## Figures and Tables

**Table 1:** Average performance of ten robots over 1000 out-of-sample episodes.

Approach	Planning Time (s)
*CommAttn*	37 ± 5
*Coordinated Frontier*	230 ± 20
*Nearest Frontier*	35 ± 5
*Cost-utility*	395 ± 15

**Table 2:** Average performance of ten robots in 30 s over 1000 out-of-sample episodes.

Approach	Average Mean Reward	Collisions	Exploration Ratio (%)
*CommAttn*	37	9 ± 6	95.2 ± 3.1
*VAIN*	21	15 ± 4	90.2 ± 3.5
*BicNet*	25	22 ± 10	89.8 ± 2.8
*CommNet*	24	13 ± 5	89.2 ± 4.1
